# Celastrol Treatment Ameliorated Acute Ischemic Stroke-Induced Brain Injury by Microglial Injury Inhibition and Nrf2/HO-1 Pathway Activations

**DOI:** 10.1155/2023/1076522

**Published:** 2023-04-11

**Authors:** Fanfan Cao, Ying Wang, Yuting Song, Fengxia Xu, Qiuhua Xie, Mei Jiang, Xinghui Liu, Denghai Zhang, Limin Xu

**Affiliations:** ^1^Sino-French Cooperative Central Lab, Gongli Hospital of Shanghai Pudong New Area, No. 207, Juye Rd., Pudong New District, Shanghai 200135, China; ^2^Ningxia Medical University, Ningxia 750000, China; ^3^Department of Clinical Laboratory, Gongli Hospital of Shanghai Pudong New Area, 207 Juye Road, Pudong New Area, Shanghai 200135, China; ^4^Department of Neurology, Gongli Hospital of Shanghai Pudong New Area, Shanghai 200135, China

## Abstract

**Background:**

Stroke is the third main reason of mortality, which is the leading reason for adult disability in the globe. Poststroke inflammation is well known to cause acute ischemic stroke- (AIS-) induced brain injury (BI) exacerbation. Celastrol (CL) has exhibited anti-inflammatory activities in various inflammatory traits though underlying mechanisms remain unknown. So, the present investigation is aimed at studying CL protective mechanism against AIS-induced BI.

**Methods:**

A mouse model regarding middle cerebral artery occlusion and an oxygen-glucose deprivation (OGD) cell model with or not CL treatment were constructed to study CL protective effects. NF-E2-related factor 2 (Nrf2) was then silenced in BV2 microglia cells (BV2) to study Nrf2 role regarding CL-mediated neuroprotection.

**Results:**

The results showed that CL treatment suppressed AIS-induced BI by inhibiting NLRP3/caspase-1 pathway activations and induction of apoptosis and pyroptosis *in vivo* and *in vitro*. NLRP3/caspase-1 pathway blocking activation suppressed OGD-induced cell pyroptosis and apoptosis. Also, CL treatment reversed OGD-induced microglial injury by promoting Nrf2/heme oxygenase-1 (HO-1) pathway activations. Nrf2 downregulation reversed CL protective effects against OGD-induced microglial injury, pyroptosis, and apoptosis.

**Conclusion:**

The findings advise that CL treatment ameliorated AIS-induced BI by inhibiting microglial injury and activating the Nrf2/HO-1 pathway.

## 1. Background

Acute ischemic stroke- (AIS-) induced brain injury (BI) and subsequent functional recovery are mediated by neuroinflammation [1]. NLR pyrin domain containing 3 (NLRP3) inflammasome is a multiprotein complex composed of ASC, NLRP3, and caspase-1 [2], which serves as a central innate immune sensor triggered by endogenous “danger” signaling in response to pathogenic infection, metabolic dysregulation, and tissue damage [3]. NLRP3 inflammasome activations enhance caspase-1 activations besides maturation and release of proinflammatory cytokines such like interleukin- (IL-) 1*β* and IL-18, causing pyroptosis promotions [4]. Pyroptosis is a proinflammatory programmed cell apoptosis process, which has distinct signaling mechanisms from those of apoptosis and necrosis [4]. So, we surmised that the suppression of pyroptosis may reverse ASI-induced BI.

Celastrol (CL), a quinone methide triterpenoid derived from *Tripterygium wilfordii*, is a traditional Chinese medicine plant, which is referred to as “Thunder God Vine.” It is reported to convey anticancer, anti-inflammatory, and antioxidative functions [5–7]. Former research found that CL can suppress expression of IL-6, IL-1, TNF-*α*, and IL-8 through suppressing NLRP3 inflammasome activations [8, 9]. Another study found that CL suppressed M1 macrophage-mediated inflammation in diet-induced obese mice through Nrf2/HO-1 pathway [10]. In addition, Nrf2/HO-1 pathway activations were shown to inhibit both inflammation and oxidative stress [11]. However, CL protective mechanism against AIS-induced BI relative to Nrf2/HO-1 pathway activation and pyroptosis promotion remains unclear.

Therefore, the current research was to investigate if CL possesses protective effects against AIS-induced BI. Briefly, NLRP3 inflammasome-induced pyroptosis and apoptosis were assessed applying *in vitro* cell model and an AIS mouse model. Results indicate that CL treatment ameliorated AIS-induced BI by inhibiting microglial injury and Nrf2/HO-1 pathway activations.

## 2. Methods

### 2.1. Animals and Ethics Statement

Male C57BL/6 background mouse (Shanghai SIPPR-Bk Lab Animal Co., Ltd., Shanghai, China) had free access to water and food under the following conditions: 22 ± 3°C temperature, 60 ± 5% humidity, and 12 h light/dark cycle. Our team treated animals following Guide for Care and Use of Laboratory Animals, and investigation protocols were made in accordance with guidelines of Ethics Committee in Pudong New Area Gongli Hospital (Shanghai, China). Surgery processes were made to minimize suffering after anesthesia.

### 2.2. Middle Cerebral Artery Occlusion (MCAO) Mouse Model

We anesthetized mouse by intraperitoneal injection with sodium pentobarbital at 30 mg/kg. We monitored body temperature to maintain it to 36.5~37.5°C. Modified MCAO-treated mouse model was utilized to induce permanent focal ischemia following [12]. In brief, we occluded right middle cerebral artery (MCA) via inserting monofilament nylon suture with 0.24~0.26 mm diameter through heat-rounded tip into internal carotid artery. It was further advanced until it closed MCA origin. Each group included a total of six mice.

### 2.3. Animal Treatments

We randomly assigned cerebral ischemia and sham-operated mice to the vehicle or CL group. Mice in the CL group were intraperitoneally injected with 1 mg/kg of CL immediately dissolved in 0.9% NaCl and 1% dimethyl sulfoxide (InvivoGen, San Diego, CA, USA) and on postoperative day 1. All mice were reanesthetized and sacrificed 3 days post MCAO.

### 2.4. Infarct Volume Measurement

Infarct volumes were analyzed following [13]. We decided infarct volume applying 2,3,5-triphenyltetrazolium chloride (TTC) at 3 d post MCAO. Our group sliced brain tissue to thick sections, which we stained with 2% solution TTC for twenty minutes at 37°C and then fixed with 4% paraformaldehyde. Our team imaged and analyzed TTC-stained sections utilizing Image-Pro Plus 5.1 (Media Cybernetics, Bethesda, MA, USA). Lesion volume is calculated as (total infarct volume + intact contralateral hemisphere volume − intact ipsilateral hemisphere volume)/contralateral hemisphere volume.

### 2.5. Histopathological Analyses

Technician fixed brain tissues in 10% buffered formalin, routinely processed, embedded in paraffin, and sliced into 4 *μ*m thick sections, which we placed on slides and stained for terminal deoxynucleotidyl transferase-mediated dUTP nick end labeling analysis (Shanghai Yeasen Biotech Co., Ltd., Shanghai, China). The slides were then evaluated under a light microscope (Motic Hong Kong Limited, Hong Kong, China).

### 2.6. BV2 Cell Culture and Transfection

BV2 cells were purchased from Wuhan Biofavor Biotechnology Service Co., Ltd. (Wuhan, China), which we maintained in DMEM (Invitrogen Corporation, Carlsbad, CA, USA) and supplied with 10% FBS (Invitrogen Corporation, Carlsbad, CA, USA) under atmosphere of 95% air/5% CO_2_. Our team seeded BV2 cells into 24-well plates with density 1 × 10^5^ cells/mL, which we incubated with serum-free DMEM over the night. On the following day, we pretreated cells with CL (1 *μ*M), MCC950 (10 *μ*g/mL), or Ac-YVAD-CMK (10 ng/mL) for 6 h in prior stimulation under OGD conditions for 3 h. Afterward, we cultured cells with normal conditions for 1 d and then collected for analysis.

Our team transfected BV2 cells with small interfering RNA against Nrf2 (GeneCopoeia, Shanghai, China) employing Lipofectamine 2000 Transfection Reagent (Invitrogen Corporation). The cells were utilized for further experiments post 48 h. The following primer sequences were used: (sense) 5′-GCA GCC AUG ACU GAU UUA A-3′ and (antisense) 5′-UUA AAU CAG UCA UGG CUG CUU-3′.

### 2.7. Apoptosis Flow Cytometric Analysis

Our team collected BV2 cells from various groups, which we resuspended in 1x binding buffer. Adding 5 *μ*L Annexin V-fluorescein isothiocyanate (FITC) solution and 5 *μ*L propidium iodide solution following protocols of Annexin V-FITC Apoptosis Detection Kit (BD Biosciences, San Jose, CA, USA), we incubated cells under room temperature for fifteen minutes in dark. Immediately afterward, we subjected cells to flow cytometry to calculate viable and apoptosis cell percentages.

### 2.8. Enzyme-Linked Immunosorbent Assay (ELISA)

IL-18, IL-1*β*, and TNF-*α* expression levels were assessed utilizing commercial ELISA kits (Abcam, Cambridge, UK) following manufacturer protocols. In brief, we collected 100 *μ*L cell culture supernatants or mouse serum, which we put to 96-well plates coated with appropriate antibody. We incubated them with enzyme conjugation solution for 60 min at 37°C. Post washing the plates five times, we added substrates I and II to the wells and incubated plates with room temperature for an additional 15 min. Finally, we terminated reactions by stopping solution addition. Our team determined absorbance through measuring optical density by microplate reader (Thermo, Waltham, MA, USA) at 450 nm wavelength.

### 2.9. Western Blot Analysis

Our team lysed cells or brain tissues in Triton X-100 lysis buffer (25 mM Tris-HCl and pH 7.5). Proteins that purified were measured by sodium dodecyl sulfate-polyacrylamide gel electrophoresis utilizing 12% separating and 5% stacking gel followed by transferred protein from the gels onto nitrocellulose membranes, which were blocked with 5% nonfat milk and TBS-T buffer (0.2% Tween 20, 150 mM NaCl, 50 mM Tris-HCl, and pH 7.5) for 1 h at room temperature. The membranes were probed with primary antibodies against Nrf2, HO-1, NLRP3, caspase-1, IL-1*β*, NF-*κ*B, and GAPDH. Following incubation with respective secondary antibodies (Santa Cruz Biotechnologies Inc., Santa Cruz, CA, USA), we incubated membranes utilizing Lumi-Phos™ WB chemiluminescent substrate (Pierce Biotechnology, Waltham, MA, USA) for 5 min. Our team measured protein expression levels via ImageJ software (NIH, Bethesda, MD, US).

### 2.10. RNA Extraction and qRT-PCR

Our team extracted total RNA utilizing TRIzol reagent (Sigma-Aldrich Corporation). We determined RNA concentrations through Epoch Microplate Spectrophotometer (BioTek Instruments, Inc., Winooski, VT, USA). Our group performed quantification of Nrf2 and endogenous control GAPDH applying TaqMan assays via supplemented assay-particular primers (Applied Biosystems, Carlsbad, CA, USA). The statistician analyzed data applying 2^−*ΔΔ*Ct^ method.

### 2.11. Statistics Analyses

We presented data by means ± SD. Statistics analyses were made via GraphPad Prism (GraphPad Software Inc., La Jolla, CA, USA) to find significant differences between groups. *p* values ≤ 0.05 were regarded as statistically significant. Two-tailed Student's *t*-tests were applied to calculate significant differences among groups, and two-way ANOVAs with post hoc Bonferroni tests were employed to determine significant differences among at least three groups.

## 3. Results

### 3.1. CL Treatment Suppressed Middle Cerebral Artery Occlusion- (MCAO-) Induced BI

**Figure 1:**
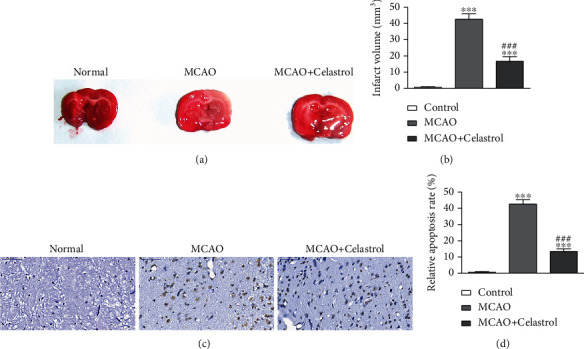
CL treatment suppressed MCAO-induced BI. (a, b) TTC staining of the infarct volume in MCAO mice with or without CL (*n* = 6). The data are presented as the mean ± SD. ^∗∗∗^*p* < 0.001 vs. control group. ^###^*p* < 0.001 vs. MCAO group. (c, d) Representative images of apoptotic cells in the infarct region of brain tissue. Data are presented as the mean ± SD. ^∗∗∗^*p* < 0.001 vs. control group. ^###^*p* < 0.001 vs. MCAO group.

Previous studies have found that CL has therapeutic and protective effects in a variety of diseases, including traumatic BI [14], cancer [15, 16], diabetes [17, 18], and myocardial ischemia [19]. Hence, the present study is aimed at determining if CL conveys a protective effect against AIS-induced BI. In a mouse model, CL was found to reverse MCAO-induced BI by decreasing the infarct volume, as determined by TTC staining (Figures 1(a) and 1(b)). Immunohistochemical staining showcased that apoptotic cell portions in brain tissue of infarct region had increased significantly after MCAO-induced AIS. Also, CL treatment decreased the rate of apoptosis in the infarct region (Figures 1(c) and 1(d)), suggesting that CL conveys a protective effect against MCAO-induced BI.

**Figure 2:**
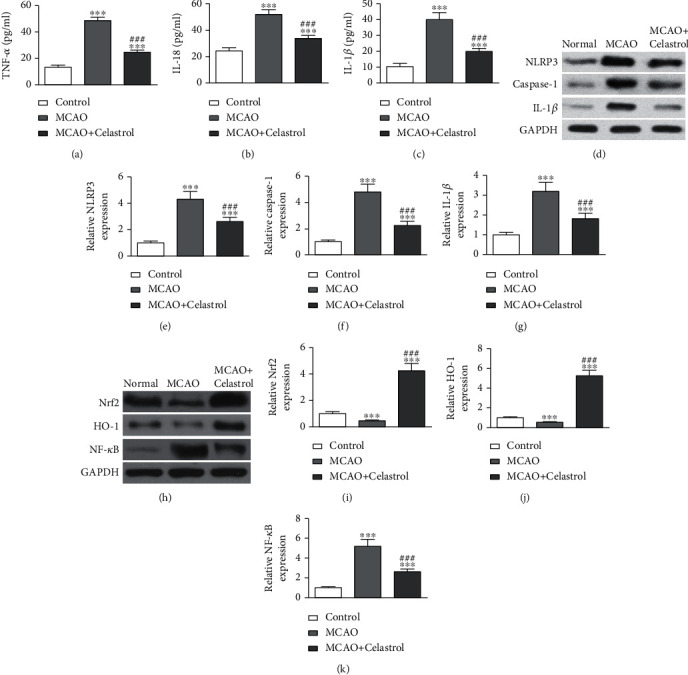
CL treatment suppressed MCAO-induced inflammatory cytokine expression. (a–c) ELISAs were used to detect the serum expression levels of TNF-*α*, IL-18, and IL-1*β*. ^∗^*p* < 0.05 and ^∗∗∗^*p* < 0.001 vs. control group. ^###^*p* < 0.001 vs. MCAO group. (d–g) Western blot detection of NLRP3, caspase-1, and IL-1*β*. The data are presented as the mean ± SD (*n* = 6). ^∗∗∗^*p* < 0.001 vs. control group. ^###^*p* < 0.001 vs. MCAO group. (h–k) Western blot detection of Nrf2, HO-1, and NF-*κ*B. The data are presented as the mean ± SD. ^∗∗∗^*p* < 0.001 vs. control group. ^###^*p* < 0.001 vs. MCAO group.

Increasing evidence has shown that CL has anti-inflammatory effect [20]. To confirm anti-inflammatory activity of CL, TNF-*α*, IL-1*β*, and IL-18 serum expression levels, they were detected with commercial ELISA kits. Data showcased that TNF-*α*, IL-18, and IL-1*β* expression levels in the MCAO group had increased, which were suppressed by CL treatment (Figures 2(a)–2(c)). Data imply that CL could suppress the inflammatory factor releases. Data of WB analyses validated that CL inhibited AIS-induced activation regarding the NLRP3 inflammasome and suppressed AIS-induced pyroptosis by reducing caspase-1 and IL-1*β* expression levels (Figures 2(d)–2(f)), suggesting that NLRP3 inflammasome activation functions importantly in pyroptosis inductions.

Former investigations found that Nrf2/HO-1 pathway functions anti-inflammatory role importantly [10, 21]. Likewise, data of the present study showcased that CL treatment promoted Nrf2/HO-1 activation and suppressed AIS-induced NF-*κ*B expression (Figures 2(g)–2(k)). These data are concordant to those of former study that Nrf2/HO-1 pathway activations suppress NLRP3 inflammasome-dependent pyroptosis [22].

### 3.2. Oxygen-Glucose Deprivation- (OGD-) Induced Microglial Injury Relative to the NLRP3/Caspase-1 Pathway Activations

**Figure 3:**
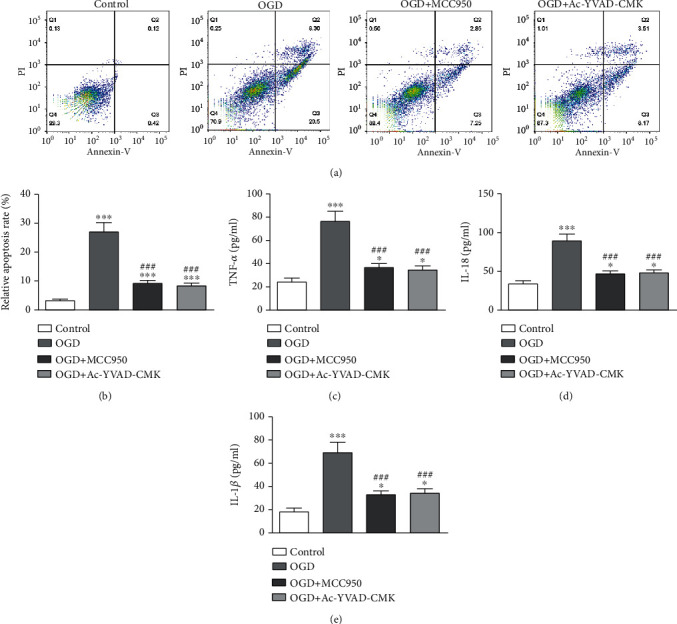
NLRP3/caspase-1 pathway plays an important role in OGD-induced microglial injury. BV2 cells were pretreated with the NLRP3 inhibitor MCC950 (10 *μ*g/mL) or the caspase-1 inhibitor Ac-YVAD-CMK (100 ng/mL) for 6 h before exposure to OGD conditions for 3 h and then cultured under normal conditions for 24 h. (a, b) Apoptosis of BV2 cells was assessed by flow cytometry using Annexin V-FITC staining, and the relative apoptosis ratio was calculated. Data are presented as the mean ± SD. ^∗∗∗^*p* < 0.001 vs. control group. ^###^*p* < 0.001 vs. OGD group. (c–e) ELISA results showing the expression levels of TNF-*α*, IL-18, and IL-1*β* in cell supernatant. Data are presented as the mean ± SD. ^∗^*p* < 0.05, ^∗∗^*p* < 0.01, and ^∗∗∗^*p* < 0.001 vs. control group. ^###^*p* < 0.001 vs. OGD group.

**Figure 4:**
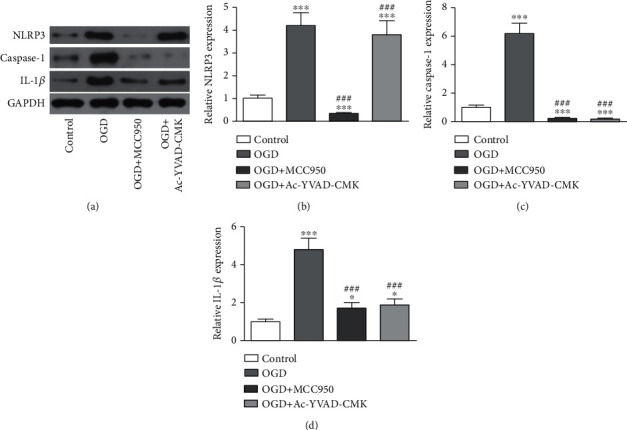
OGD-induced microglial injury relative to activation of the NLRP3/caspase-1 pathway. (a–d) Western blot detection of NLRP3, caspase-1, and IL-1*β*. The data are presented as the mean ± SD. ^∗^*p* < 0.05, ^∗∗^*p* < 0.01, and ^∗∗∗^*p* < 0.001 vs. control group. ^###^*p* < 0.001 vs. MCAO group.

To determine if NLRP3 inflammasome and caspase-1 are involved in AIS-induced BI, BV2 cells were cultured under OGD conditions to simulate AIS in an *in vitro* cell model as previously reported [23]. The result showed that the portion of apoptotic BV2 cells had increased under OGD conditions for 3 h. But pretreatment with NLRP3 inhibitor MCC950 or caspase-1 inhibitor Ac-YVAD-CMK decreased apoptotic BV2 cell portions (Figures 3(a) and 3(b)). ELISA results showed that blocking activation of NLRP3/caspase-1 pathway suppressed OGD-induced IL-18, IL-1*β*, and TNF-*α* expressions (Figures 3(c)–3(e)). WB data showcased that NLRP3/caspase-1 pathway activations blocked the decrease in IL-1*β* expression (Figures 4(a)–4(d)).

### 3.3. CL Treatment Reversed OGD-Induced Microglial Injury by HO-1/Nrf2 Pathway Activations

**Figure 5:**
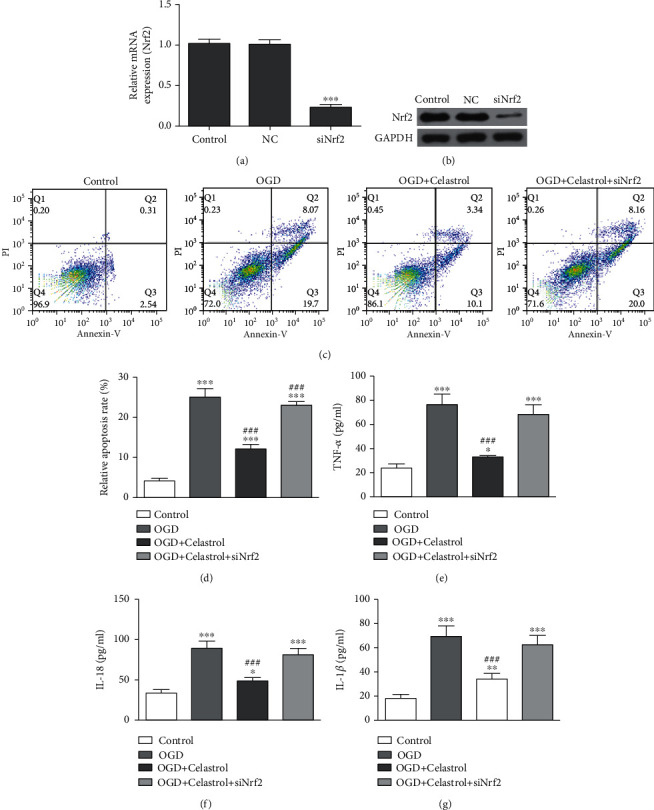
CL treatment reversed OGD-induced microglial injury. BV2 cells with or without Nrf2 silencing were pretreated with CL (1 *μ*M) for 6 h before exposure to OGD conditions for 3 h and then cultured under normal conditions for 24 h. (a, b) RT-PCR and western blot detection of Nrf2 at the mRNA and protein levels, respectively. Data are presented as the mean ± SD. ^∗∗∗^*p* < 0.001 vs. control group. (c, d) Apoptosis of BV2 cells was assessed by flow cytometry using Annexin V-FITC staining, and the relative apoptosis ratio was calculated. Data are presented as the mean ± SD. ^∗∗∗^*p* < 0.001 vs. control group. ^###^*p* < 0.001 vs. OGD group. (e–g) ELISA results showing the expression levels of TNF-*α*, IL-18, and IL-1*β* in cell supernatant. Data are presented as the mean ± SD. ^∗^*p* < 0.05, ^∗∗^*p* < 0.01, and ^∗∗∗^*p* < 0.001 vs. control group. ^###^*p* < 0.001 vs. OGD group.

**Figure 6:**
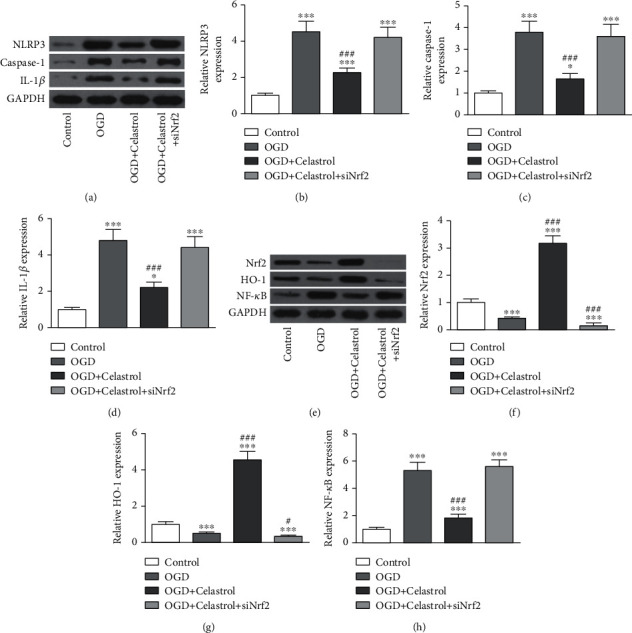
CL treatment reversed OGD-induced microglial injury by promotion of the Nrf2/HO-1 pathway. (a–d) Western blot detection of NLRP3, caspase-1, and IL-1*β*. The data are presented as the mean ± SD. ^∗^*p* < 0.05 and ^∗∗∗^*p* < 0.001 vs. control group. ^###^*p* < 0.001 vs. OGD group. (e–h) Western blot detection of Nrf2, HO-1, and NF-*κ*B. The data are presented as the mean ± SD. ^∗∗∗^*p* < 0.001 vs. control group. ^#^*p* < 0.05 and ^###^*p* < 0.001 vs. OGD group.

In order to decide if CL treatment reversed OGD-induced microglial injury through Nrf2/HO-1 pathway activations, Nrf2 silencing in BV2 cells was conducted, which showed that silencing of Nrf2 significantly decreased Nrf2 expression at protein and mRNA levels (Figures 5(a) and 5(b)). Furthermore, flow cytometry data showcased that OGD induction promoted BV2 cell apoptosis, which was reversed by CL treatment. However, the protective effect of CL decreased after downregulation of Nrf2 (Figures 5(c) and 5(d)). Our data advise that Nrf2 functions importantly in CL-mediated neuroprotection in AIS. Meanwhile, ELISA data showcased that Nrf2 downregulation reversed CL anti-inflammatory effect (Figures 5(e)–5(g)). WB data showcased that CL treatment suppressed OGD-induced NLRP3/caspase-1/IL-1*β*-mediated cell pyroptosis, but downregulation of Nrf2 reversed the protective effect of CL (Figures 6(a)–6(d)) and suppressed HO-1 expression, which caused NF-*κ*B expression upregulations under OGD conditions even with CL treatment (Figures 6(e)–6(h)).

## 4. Discussion

The present study showed that NLRP3/caspase-1 pathway participated in AIS-induced apoptosis and pyroptosis, whereas NLRP3/caspase-1 pathway inhibitions suppressed AIS-induced BI. Also, caspase-1, NLRP3, and IL-1*β* expression levels constitute inflammasome pathway. We monitored IL-1*β*, caspase-1, and IL-18 activations in serum and cell supernatant. Results advised that NLRP3 inflammasome activations promoted microglial pyroptosis. Also, NLRP3 functions in innate immune responses to pathogenic infection and metabolic stress relevant to systemic traits, such as diabetes and sepsis, as well as atherosclerotic lesion and stroke formations [24–28]. So, we surmised that inhibiting NLRP3 inflammasome-mediated pyroptosis will suppress AIS-induced nerve injury.

The data also showed that CL treatment reversed AIS- and OGD-induced nerve injury by suppressing NLRP3/caspase-1 pathway activations. CL was widely applied for various autoimmune disease treatments [29–31]. Previous investigations showcased that CL suppressed NLRP3 inflammasome-mediated IL-18 and IL-1*β* release, along with pyroptosis activation [9]. Meanwhile, CL treatment reversed AIS-induced BI by microglial injury inhibition and Nrf2/HO-1 pathway activations, whereas downregulation of Nrf2 reversed CL protective effect. HO-1/Nrf2 activation suppressed oxidative stress and inflammatory cytokine productions by decreasing NF-*κ*B expressions. Nrf2 is a main factor in cytoprotection, which activated under stress conditions resulted from electrophilic and reactive oxygen species productions. Inflammasomes are central inflammation regulators [32]. Dihydromyricetin suppresses NLRP3 inflammasome-dependent pyroptosis by Nrf2 signaling pathway activations in vascular endothelial cells [22]. Outputs of current investigations showcased that CL treatment ameliorated AIS-induced BI by inhibiting microglial injury and Nrf2/HO-1 pathway activations. Previous investigations advised that celastrol can be used as novel drug to cause cerebral vascular dilation in cases where endothelial and/or BK channel function is impaired [33]. But if CL treatment can improve the vascular system after acute ischemic stroke is still unclear.

## 5. Conclusions

**Figure 7:**
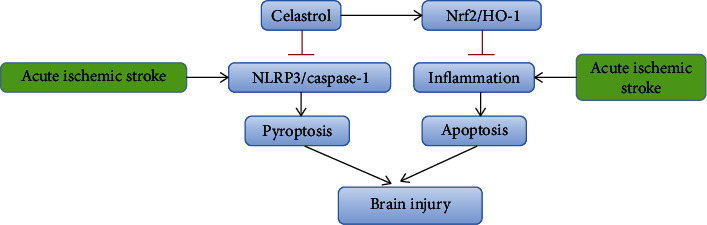
The regulatory mechanism of celastrol. CL treatment inhibits acute ischemic stroke-induced and NLRP3/caspase-1 pathway-mediated pyroptosis. At the same time, CL treatment promoted Nrf2/HO-1 pathway, which inhibit inflammatory cytokine-mediated apoptosis.

Our results highlight the pyrogenic properties of NLRP3 inflammatories and the antipyrogenic properties of CL, as well as their role in the pathogenesis of AIS-induced BI. Our study found that CL treatment inhibited acute ischemic stroke-induced and NLRP3/caspase-1 pathway-mediated pyroptosis. Meanwhile, CL treatment promoted Nrf2/HO-1 pathway and inhibited apoptosis mediated by inflammatory cytokines (Figure 7). The data may have indispensable implications for new therapeutic strategy developments to treat this severe AIS complication.

## Data Availability

The data used to support the findings of this study are available from the corresponding authors upon request.
